# Construction of an acute myeloid leukemia prognostic model based on m6A-related efferocytosis-related genes

**DOI:** 10.3389/fimmu.2023.1268090

**Published:** 2023-11-22

**Authors:** Ying Wang, Ting Bin, Jing Tang, Xiao-Jun Xu, Chao Lin, Bo Lu, Tian-Tian Sun

**Affiliations:** ^1^ Department of Haematology. The Seventh Affiliated Hospital, Sun Yat-Sen University, Shenzhen, China; ^2^ Pediatric Hematology Laboratory, Division of Hematology/Oncology, Department of Pediatrics. The Seventh Affiliated Hospital, Sun Yat-Sen University, Shenzhen, China

**Keywords:** acute myeloid leukemia, N6-methyladenosine, efferocytosis, prognostic risk model, bioinformatics, drug prediction

## Abstract

**Background:**

One of the most prevalent hematological system cancers is acute myeloid leukemia (AML). Efferocytosis-related genes (ERGs) and N6-methyladenosine (m6A) have an important significance in the progression of cancer, and the metastasis of tumors.

**Methods:**

The AML-related data were collected from The Cancer Genome Atlas (TCGA; TCGA-AML) database and Gene Expression Omnibus (GEO; GSE9476, GSE71014, and GSE13159) database. The “limma” R package and Venn diagram were adopted to identify differentially expressed ERGs (DE-ERGs). The m6A related-DE-ERGs were obtained by Spearman analysis. Subsequently, univariate Cox and Least Absolute Shrinkage and Selection Operator (LASSO) were used to construct an m6A related-ERGs risk signature for AML patients. The possibility of immunotherapy for AML was explored. The pRRophetic package was adopted to calculate the IC50 of drugs for the treatment of AML. Finally, the expression of characterized genes was validated by quantitative reverse transcription-PCR (qRT-PCR).

**Results:**

Based on m6A related-DE-ERGs, a prognostic model with four characteristic genes (UCP2, DOCK1, SLC14A1, and SLC25A1) was constructed. The risk score of model was significantly associated with the immune microenvironment of AML, with four immune cell types, 14 immune checkpoints, 20 HLA family genes and, immunophenoscore (IPS) all showing differences between the high- and low-risk groups. A total of 56 drugs were predicted to differ between the two groups, of which Erlotinib, Dasatinib, BI.2536, and bortezomib have been reported to be associated with AML treatment. The qRT-PCR results showed that the expression trends of DOCK1, SLC14A1 and SLC25A1 were consistent with the bioinformatics analysis.

**Conclusion:**

In summary, 4 m6A related- ERGs were identified and the corresponding prognostic model was constructed for AML patients. This prognostic model effectively stratified the risk of AML patients.

## 1 Introduction

Acute myeloid leukemia (AML) refers to a kind of malignant disease originated from haemopoietic stem cells and features clonal expansion of blasts of myeloid lineage under abnormal differentiation. Upon the proliferation of immature myeloid cells, immature progenitors (blasts) are accumulated and normal hemopoiesis is impaired, resulting in the occurrence of serious infection, anemia and hemorrhage (1). AML accounts for 1.3% of newly diagnosed cancer cases in the U.S (2). The rising AML incidences are partially a result of rising prevalence of AML related to therapy, because the primary malignancy of increasing numbers of cancer patients who received cytotoxic chemotherapy have been cured (3). AML is one of the most fatal type of hematologic malignancy, with the 5-year survival rate < 30%. Hence, new prognostic biomarkers shall be urgently identified for well monitoring patient outcomes as well as more deeply explaining the AML pathogenesis.

Recent researchers have identified reversible N6-methyladenosine (m6A) RNA methylation regulators as a new way to achieve the post-transcriptional regulation (4). Geneticists confirmed that m6A was methylated in eukaryotic messenger RNA (mRNA). RNA methylation modification takes up over 60% of all the RNA modifications, of which the representative type on higher biological mRNAs is the m6A RNA methylation (5). When m6A regulators are dysregulated, the cell reproductive capacity weakens, and the self-renewal capacity losses, together with developmental defects and apoptosis (6). m6A RNA methylation regulators play roles in the cancer occurrence and development, involving liver cancer (7, 8), glioblastoma (9), osteosarcoma (10), and colorectal cancer (11).

Macrophages critically impact the remodeling of tissues in normal physiology and the way inflammation and tissue injury are resolved (12). The key step in the resolution process lies in the elimination of apoptotic cells (ACs) (13). The elimination of apoptotic cells by professional and non-professional phagocytes, a process that is essential for maintaining tissue homeostasis called “efferocytosis” (13, 14). Such process has been informed in recent studies, together with the roles it plays in maintaining tissue homeostasis and repair as well as the organism health. Our study stresses on the mechanisms regarding efferocytosis (dying cell recognition, phagocytic engulfment and homeostatic resolution), as well as explains the resulting pathological and physiological consequences upon the abrogation of the efferocytosis process (13). As we all know, m6A regulates gene expression and thus cellular processes such as cellular self-renewal, differentiation, invasion, and apoptosis (15). For example, Mettl14-mediated m6A modification could induce apoptosis of spinal cord neurons in spinal cord injury by promoting miRNA translation (16). Apoptosis and its subsequent clearance by efferocytosis occur in virtually all tissues during development, homeostasis, and disease. However, the prognostic value of m6A-related efferocytosis related-genes (ERGs) in AML has not been systematically investigated.

AML is an aggressive blood cancer among adults, and the existing techniques fail to obviously improve most patients’ survival rate. In the situation, it is necessary to find potential markers for enhancing AML patients’ diagnosis, treatment and prognosis. According to previous studies, we inferred m6A RNA methylation regulators and efferocytosis were inextricably linked to the onset and progression of AML. Our study adopts AML-related data from public database and the comprehensive biological informatics approach for mining the m6A-related ERGs in AML. The prognostic model related to m6A and ERGs was constructed to predict the prognosis of the AML patients. In addition, the exploration of potential molecular mechanisms and therapeutic approaches will contribute to the treatment and prognosis of AML.

## 2 Materials and methods

### 2.1 Acquirement of the data of the AML patients

Bone marrow samples of 132 TCGA-AML (Illumina platform) patients were downloaded from the Cancer Genome Atlas (TCGA) (https://portal.gdc.cancer.gov/), and these AML patients with complete clinical information and survival data were used as the training set for the follow-up analysis. Three independent cohorts (GSE9476, GSE71014, and GSE13159) were acquired from the Gene Expression Omnibus (GEO) database (https://www.ncbi.nlm.nih.gov/geo/). The GSE71014 dataset (Illumina HumanHT-12 V4.0 expression beadchip) containing the RNA-seq and survival data of 104 AML patients was used as the validation set. The GSE9476 dataset (Affymetrix Human Genome U133A Array) contains 10 normal samples and 7 AML bone marrow samples for differential expression analysis. Peripheral blood samples from the GSE13159 dataset (Affymetrix Human Genome U133 Plus 2.0 Array) were excluded, and 73 normal samples and 501 AML bone marrow samples were obtained for expression validation of characteristic genes. Based on the previous paper, 74 efferocytosis-related genes (ERGs) were acquired (17, 18).

### 2.2 Identification of m6A -related differentially expressed ERGs

The data annotations in the GSE9476 dataset were performed based on the Symbol conversions corresponding to the chips in the GPL96 file. The raw count is converted to FPKM mainly by the following formula:


$$\text{FPKM}=\text{ExonMappedFragments}*10^{9}/\text{TotalMappedFragments}*\text{ExonLength}$$

CEL files were generated using MAS 5.0 software (Affymetrix) with target signals for probe sets scaled to 500. Log2 expression values for individual probe sets were generated from. CEL files via robust multi-array average (gcRMA). The “limma” package, a package for analyzing gene expression data generated by microarray or RNA-seq technology (19), was used to obtain the DEGs in the GSE9476 dataset. The screening criteria was: p value < 0.05 and |log_2_FoldChange| > 0.5 (20, 21). The “VennDiagram” package (22) was applied to visualize the differentially expressed ERGs (DE-ERGs). Spearman and Wilcoxon.test were applied to screen for m6A-related DE-ERGs (|cor| > 0.3 and p < 0.05). Prediction of miRNAs for m6A-related DE-ERGs was performed using TargetScan, miRTarBase, and starBase databases. Based on lncbase, starbase, and miRNet database, lncRNAs were subsequently predicted based on miRNAs common to the three databases. Cytoscape software was adopted to visualize the lncRNA-miRNA-mRNA network.

### 2.3 Acquirement of m6A-related DE-ERGs-related subtypes in the training set

Consensus Clustering, an unsupervised clustering method, can divide samples into subtypes based on different datasets, resulting the discovery of new disease subtypes. The R package “ConsensusClusterPlus” (23) was utilized to identify the subtypes based on the expression of m6A -related DE-ERGs. Additionally, the overall survival (OS) among different subtypes was explored by the “Survival” package (24). Enrichment pathways for inter-subtype differences were assessed using Gene Set Variation Analysis (GSVA) (25). The Cell-type identification by estimating relative subsets of RNA transcripts (CIBERSORT) algorithm (26) was utilized to analyze the abundance of immune cell infiltration for all samples between the subtypes. To examine the variations in immune cells between two subtypes, the Wilcoxon test was used.

### 2.4 Constructing a new AML prognostic risk model based on m6A -related DE-ERGs

The data of TCGA-AML were transformed based on the hg38 human reference genome. After transforming the data in the form of count into the form of FPKM, the FPKM was then log_2_(fpkm+1) computed to get the final FPKM value, which was our normalization method. By using univariate Cox analysis of m6A -related DE-ERGs in the TCGA-AML dataset, the prognosis-related genes were acquired (P < 0.05). Subsequently, the most predictive characteristic genes were identified by the least absolute shrinkage and selection operator (LASSO) (27). Subsequently, the risk score of each AML patient was calculated based on the formula:


$$\text{Riskscore}=\sum_{1}^{n}coef\left(gene_{i}\right)*expr\left(gene_{i}\right)$$

Based on the median risk score, the AML patients were divided into two groups. The difference in OS between the two groups was then displayed using Kaplan-Meier (KM) curves. The “survROC” (28) was applied to display the receiver operating characteristic (ROC) curves to perform an assessment of the prognostic capability of the prognostic risk model. At last, the stability of this prognostic risk model was investigated in the external GSE71014 dataset. Meanwhile, the wilcoxon test was applied to evaluate the expression of characteristic genes in the training and validation sets.

### 2.5 Assessment of prognostic risk model

In order to explore the differences in biological functions between high- and low-risk groups, we performed Gene Set Variation Analysis (GSVA). The “GSVA” package (25) was used to calculated the score of the pathways in samples, and “limma” package (19) was used to implement the differential analysis of pathways (|t value| > 2). Since somatic mutations play a critical role in tumor development, we investigated the tumor body mutations of samples in the two groups by “maftools” package (29), and showed the top20 mutated genes, respectively. Clinicopathological characteristics of TCGA-AML included cytogenetic risk, age, M subtype, bone marrow (BM) blasts (%), invasiveness, and Platelets (x10^9/L). To determine if clinicopathological characteristics and risk scores were independent predictive factors for AML patients, univariate and multifactorial Cox analyses were performed. The “rms” (30) was adopted to construct the nomogram to predict survival probability based on independent prognostic criteria. The calibration curve was adopted to validate whether the nomogram has good predictive power.

### 2.6 Relationship between AML patients’ risk scores and tumor microenvironment

The “estimate” package (31) was adopted to compare the stroma, immune, estimate score, and tumor purity between the high/low-risk groups. Spearman’s rank correlation was used to analyze the correlation between TME and risk score. The CIBERSORT (32) was utilized to analyze the abundance of immune cell infiltration for all samples in the TCGA-AML dataset. To examine the variations in immune cells between the two groups of AML, the Wilcoxon test was used. Subsequently, 48 immune checkpoint and HLA family genes were analyzed for differences in expression between the two groups. The Cancer Immunome Atlas framework (https://www.tcia.at/home) was adopted to calculate immunophenoscore (IPS) of each TCGA-AML patient sample. IPS predicts patient response to immunotherapy, with higher scores associated with greater immunogenicity (33). Calculation of mRNA expression-based stemness index (mRNAsi) scores of TCGA-AML patients was performed by “glmnet” package (34), and then the correlation between mRNAsi scores and risk scores was analyzed by Spearman.

### 2.7 Prediction of chemotherapy drug

Using the “pRRophetic”, the chemotherapy medicines for AML were predicted based on GDSC (https://www.cancerrxgene.org/) (35). To compare the two groups’ differences in drug sensitivity, we adopted the Wilcoxon.test.

### 2.8 Validation of expression of characterized genes

The AML and normal samples were collected form Seventh Affiliated Hospital, Sun Yat-Sen University according to the following inclusion criteria: (1) aged 18-60 years; (2) bone marrow; (3) initial diagnosed AMI patients and healthy donor. The exclusion criteria of AMI samples were M3 and therapeutic interventions (such as chemotherapeutic agents), and bone marrow stimulated by granulocyte colony-stimulating factor. The clinical information of AML and normal samples in qRT-PCR (**Supplementary Table 1**). This study was approved by Sanming Project of Medicine in Shenzhen (No.SZSM201911004), Ethics Committee of Seventh Affiliated Hospital, Sun Yat-Sen University. The expression of characterized genes was verified using quantitative reverse transcription-polymerase chain reaction (qRT-PCR). Total RNA was extracted from bone marrow samples (6 AML samples and 5 normal samples) with TRIzol method. The reverse transcription reactions were performed using SureScript-First-strand-cDNA-synthesis-kit (Servicebio, China), and then used to perform qRT-PCR with Universal Blue SYBR Green qPCR Master Mix. The qRT-PCR thermocycling protocol was as follows: initial denaturation at 95°C for 60 s, denaturation at 95°C for 20 s, annealing at 55°C for 20 s, extension 72°C for 30s, and amplification for 40 cycles. GAPDH was used as the housekeeping gene. The primer sequences were shown in **Table 1**. The 2^-△△CT^ method was applied to calculate the expression level of genes and normalized to GAPDH.

**Table 1:** The primer sequences of characteristic genes.

Primers	Sequence
DOCK1 F	GTTTGCTGCAACCCCTTCTCT
DOCK1 R	GACCAGCGAACCAGGTAGT
SLC14A1 F	TGGCTGTTACTCCCTGTATGTGC
SLC14A1 R	ATGGATTGTAATGTCCTGTGGC
SLC25A1 F	CCGTCAGGTTTGGAATGTTCG
SLC25A1 R	TAACCCCGTGGAAGAATCCTC
UCP2 F	GGAGGTGGTCGGAGATACCAA
UCP2 R	ACAATGGCATTACGAGCAACAT
GAPDH F	CGAAGGTGGAGTCAACGGATTT
GAPDH R	ATGGGTGGAATCATATTGGAAC

## 3 Result

### 3.1 The m6A-related DE-ERGs for AML

**Figure 1:**
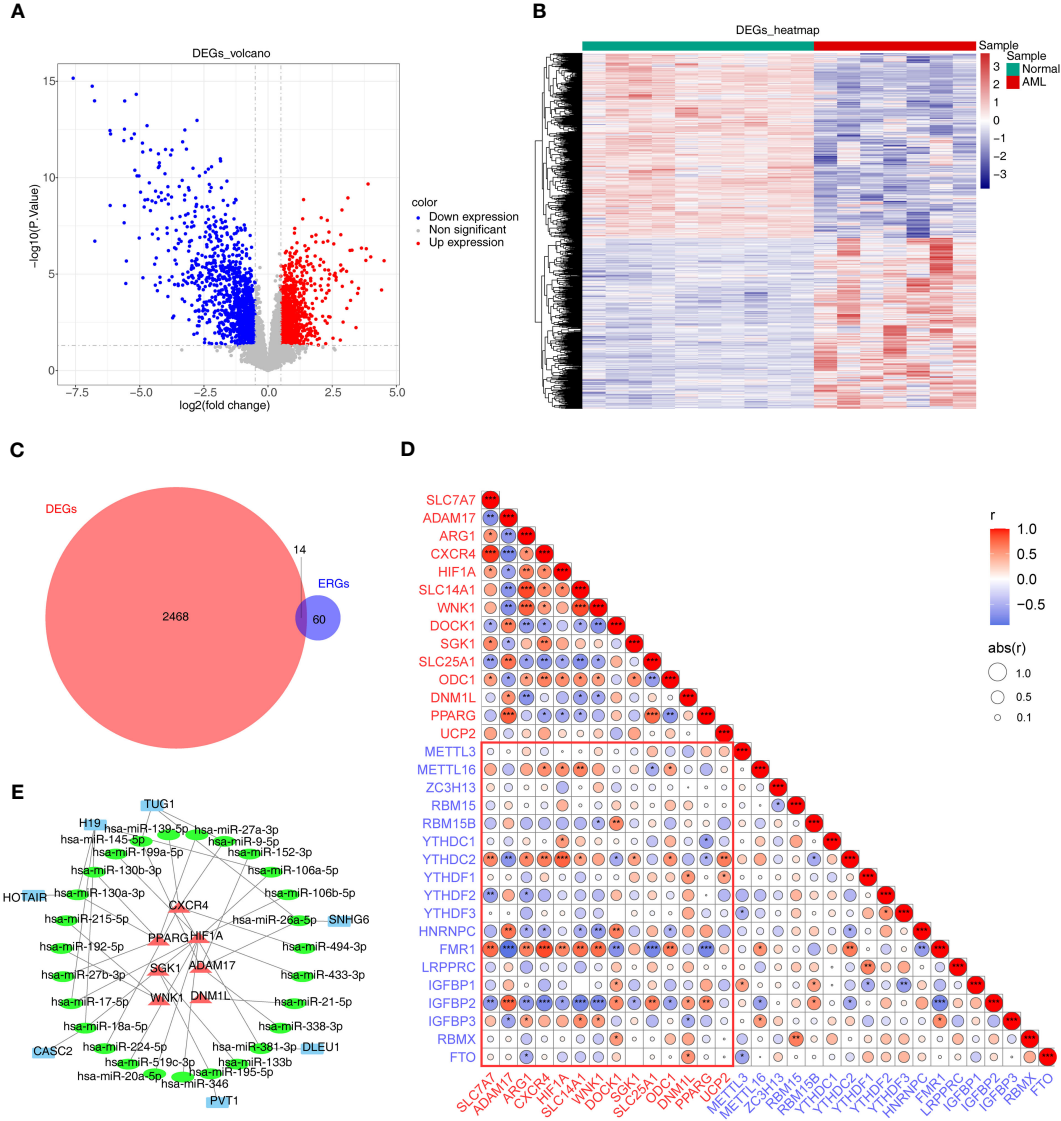
Differential expression analysis. **(A, B)** The volcano map **(A)** and heat map **(B)** of up- and down-regulated DEGs. **(C)** The Venn diagram of 14 DE-ERGs obtained by overlapping ERGs and DEGs. **(D)** The relevance of DE-ERGs and m6A-related genes. Genes in red text are DE-ERGs and genes in blue text are m6A regulators. The color and size of the circles indicate the direction and size of the correlation. *p<0.05; ** p<0.01; ***p<0.001. **(E)** The network constructed based on m6A-DE-ERGs, miRNAs, and lncRNAs. Red represents m6A-DE-ERGs, green represents miRNAs, and blue represents lncRNAs. DEGs, differentially expressed genes; AML, acute myeloid leukemia; ERGs, efferocytosis-related genes; DE-ERGs, differentially expressed ERGs; miRNA, microRNA; lncRNA, long non-coding RNA.

There were 2482 DEGs (up=1192 and down=1290) (**Figures 1A, B**) and a total of 14 ERGs were differentially expressed in the GSE9476 dataset (**Figure 1C**). And then 14 m6A related-DE-ERGs were obtained by Spearman’s correlation analysis (**Figure 1D**). A total of 27 predicted miRNAs based on 14 m6A-associated DE-ERGs were common across the 3 databases (**Supplementary Table 2**). Subsequently, based on shared miRNAs, 7 of the predicted lncRNAs were common across the 3 databases (**Supplementary Table 3**). Finally, 7 m6A-DE-ERGs, 27 miRNAs, and 7 lncRNAs of the lncRNA-miRNA-mRNA network were constructed (**Figure 1E**). It was known from the network that DLEU1 could only affect HIF1A by regulating hsa-miR-381-3p, while HIF1A could be affected by multiple miRNAs.

### 3.2 The m6A-related ERGs related-subtypes for AML

**Figure 2:**
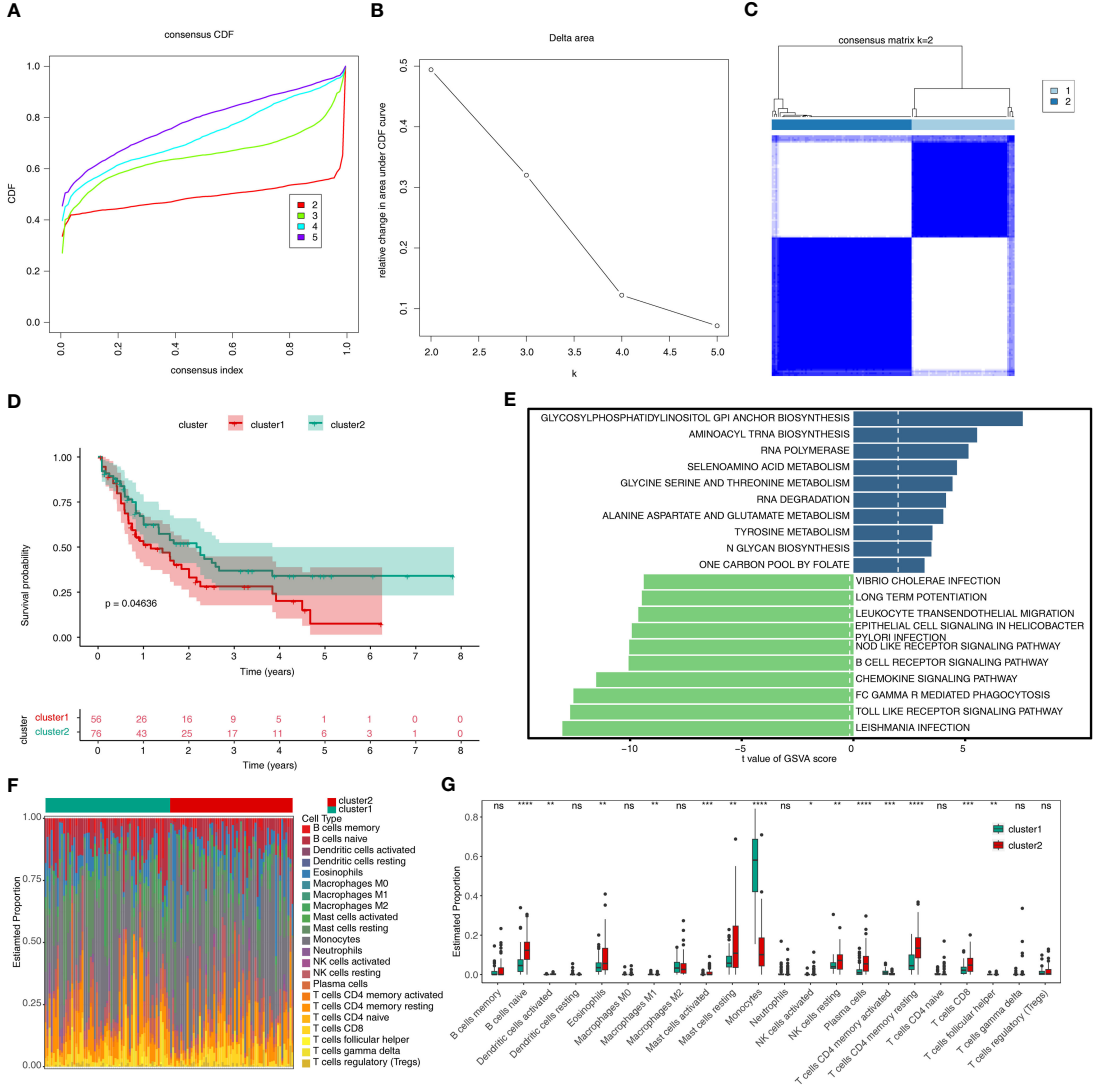
Results of consensus clustering of 132 AML patients. **(A)** Consensus clustering CDF for k = 2 to k = 5. **(B)** The corresponding relative change in area under the CDF curves when cluster number changed from k to k + 1. **(C)** Consensus clustering matrix of 132 AMI samples for k = 2. **(D)** The survival difference between cluster1 and cluster2, which are shown below the survival graph, are the number of samples corresponding to that survival time. **(E)** The top10 up- and down-regulated pathways enriched in two sub-types. **(F)** The heat map of 22 immune cells in cluster1 and cluster2. **(G)** Discrepancies of the proportion of immune cells in two sub-types. CDF, cumulative distribution function; GSVA, Gene Set Variation Analysis. ns, not significant; *p<0.05; ** p<0.01; ***p<0.001; ****p<0.0001.

Based on the expression of 14 m6A related-DE-ERGs, 132 AML patients were classified into two subtypes (**Figures 2A–C**). The cluster1 had a worse prognosis than cluster2 (p < 0.05) (**Figure 2D**). **Figure 2E** showed that ERGs associated with m6A may be regulated through various amino acid metabolic pathways and some down-regulated pathways (chemokine signaling pathway, B-cell receptor signaling pathway, and Fc gamma R-mediated phagocytosis). 22 immune cells were present in some abundance between the two subtypes (**Figure 2F**). **Figure 2G** revealed that 14 immune cell types were differentially expressed between the two subtypes, of which the proportion of naive B cells, eosinophils, resting mast cells, resting NK cells, plasma cells, resting CD4 memory T cells, and CD8 T cells was significantly higher in cluster 2 than cluster 1. Moreover, the proportion of monocytes was notably higher in the cluster 1. In summary, we speculated that the activity in terms of immune response, immune surveillance, and cellular immunity was stronger in cluster 2, whereas the increase in the proportion of monocytes may imply that the effects of immunomodulation are stronger in cluster 1.

### 3.3 The m6A-related ERGs prognostic risk model for AML

**Figure 3:**
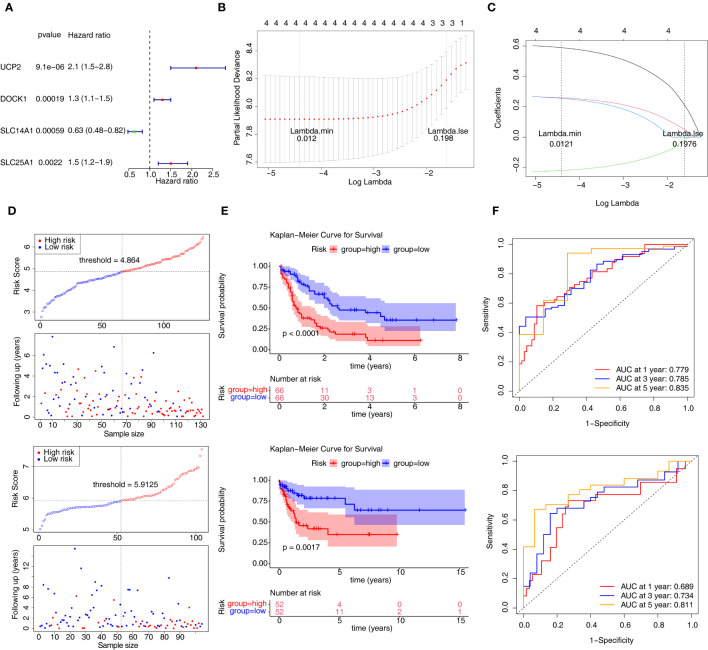
Construction and validation of the prognostic risk model. **(A)** Univariate Cox analysis of four genes. **(B, C)** The error plots for 10-fold cross-validation **(B)** and the plot of gene coefficients **(C)** in least absolute shrinkage and selection operator (LASSO) analysis. Each line **(C)** represents a gene. **(D)** The risk curve of prognostic risk model in the training set and GSE71014 dataset. **(E)** The Kaplan-Meier curves of high- and low-risk groups in two datasets. **(F)** The ROC curves of 1/3/5-year in the training set and GSE71014 dataset. ROC, receiver operating characteristic; AUC, area under the curve.

According to m6A related-DE-ERGs, four genes with p < 0.05 were screened in the training set (**Figure 3A**). After that, LASSO regression analysis was carried out to obtain four characteristic genes (UCP2, DOCK1, SLC14A1, and SLC25A1) (**Figures 3B, C**). Risk score = 0.5890×UCP2 + 0.2590×DOCK1 -0.2193×SLC14A1 + 0.2553×SLC25A1. Based on median risk score = 4.864, patients were classified into two groups (**Figure 3D**). Patients with low-risk scores had significantly higher OS than those with high-risk scores (**Figure 3E**). The ROC curve for OS was computed to further evaluate the validity of the risk signature, and the AUC values at 1, 3, and 5 years were larger than 0.70, demonstrating improved efficacy of the prognostic risk model (**Figure 3F**). The prognostic risk model still had strong predictive power in the GSE71014 datasets (**Figures 3D–F**). The expression trends of DOCK1 and SLC25A1 were increased in AML, while the opposite was true for SLC14A1 and UCP2 in the GSE9476 and GSE13159 datasets (**Supplementary Figure S1**).

### 3.4 The biological and mutational changes in AML

**Figure 4:**
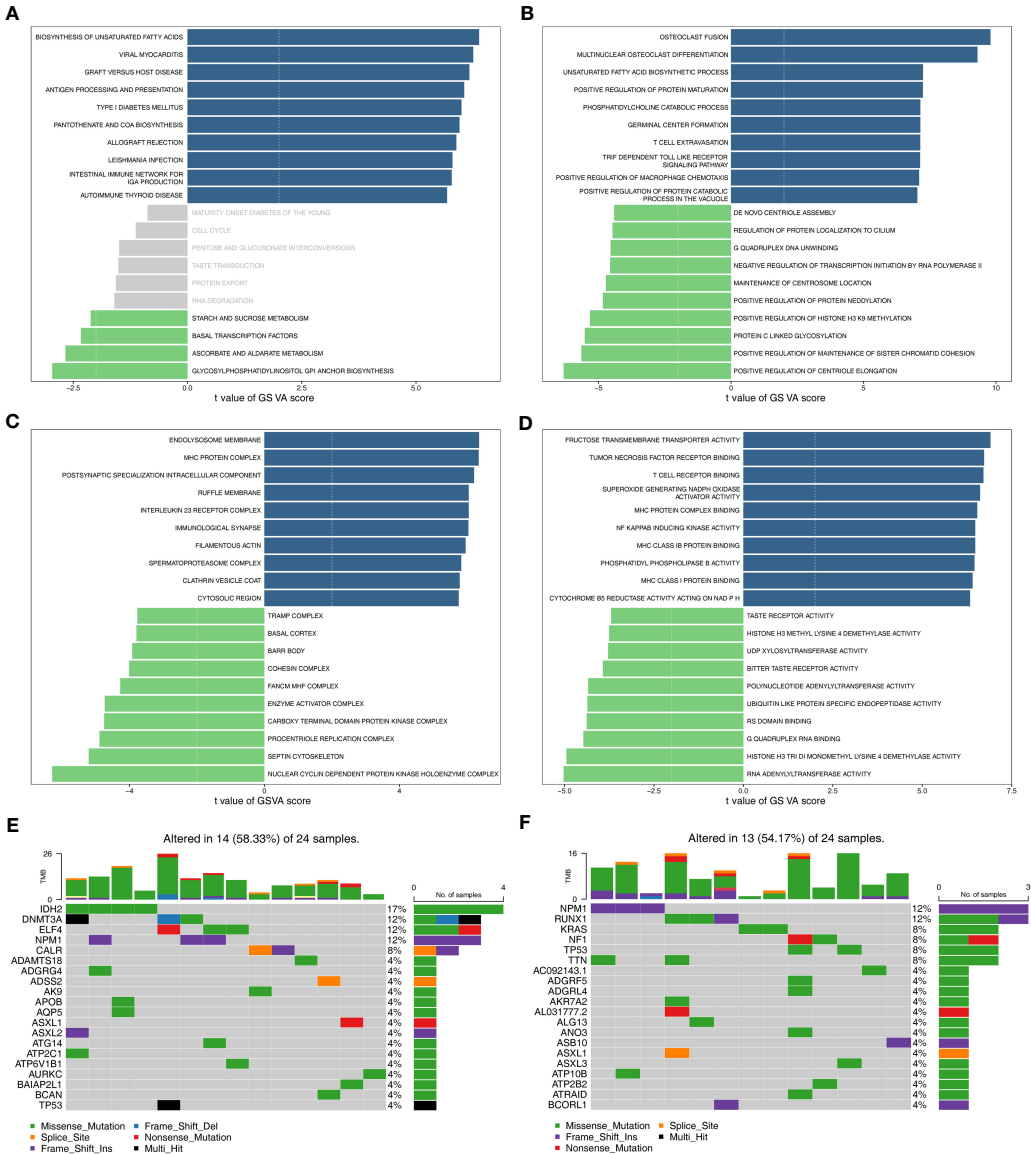
Functional enrichment analysis and somatic mutation analysis. **(A)** The KEGG pathways enriched in high- and low-risk groups. **(B–D)** The GO terms enriched in two risk groups. B: BP; C: CC; D: MF. **(E)** Top 20 genes with the highest mutation frequency in the high-risk group. **(F)** Top 20 genes with the highest mutation frequency in the low-risk group. GO, gene ontology; KEGG, Kyoto Encyclopedia of Genes and Genomes; BP, biological progress; CC, cellular component; MF, molecular function.

A total of 4083 GO entries and 117 pathways now differ between the high/low-risk groups (|t|>2). **Figure 4A** displayed the top10 up-regulated and top4 down-regulated KEGG pathways that were significantly different between high- and low-risk groups, e.g., biosynthesis of unsaturated fatty acids, antigen processing and presentation, and pantothenate and CoA biosynthesis. Moreover, the top10 up- and down-regulated GO terms (including biological progress (BP), cellular component (CC), and molecular function (MF)) that were notably different between two risk groups were shown in **Figures 4B–D**. Interestingly, some immune-related biological functions, such as T cell extravasation, MHC protein complex, T cell receptor binding, and MHC class I protein binding, were significantly up-regulated in GO terms that differed significantly between high- and low-risk groups. **Figures 4E, F** showed the top20 mutated genes in the high- and low-risk groups, of which ASXL1, NPM1, and TP53 were mutated between both groups.

### 3.5 The independent predictors and nomogram in AML

**Figure 5:**
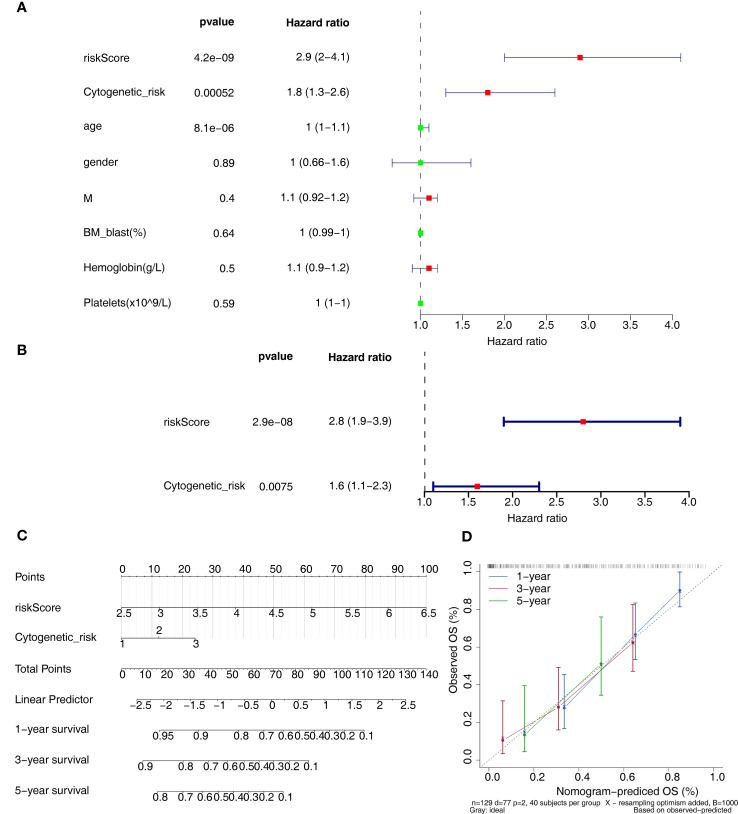
Independent prognostic analysis. **(A, B)** The independent prognostic predictors obtained by univariate **(A)** and multivariate **(B)** Cox analyses. **(C)** The nomogram of risk score and cytogenetic risk. **(D)** The calibration curve of the nomogram.

Clinicopathological variables and risk scores from 132 patients were combined to perform univariate and multivariate Cox analyses (**Figures 5A, B**). The risk scores and Cytogenetic risk was the prognostic factor for AML patients. Construction of a nomogram model on the basis of independent prognostic factors (**Figure 5C**), it was found that the survival rate decreases as the overall score increases. The slope of the calibration curve of the model is close to 1, indicating that the predictions of the model are true and reliable (**Figure 5D**).

### 3.6 The differences of immune microenvironment and immunotherapy between two risk groups

**Figure 6:**
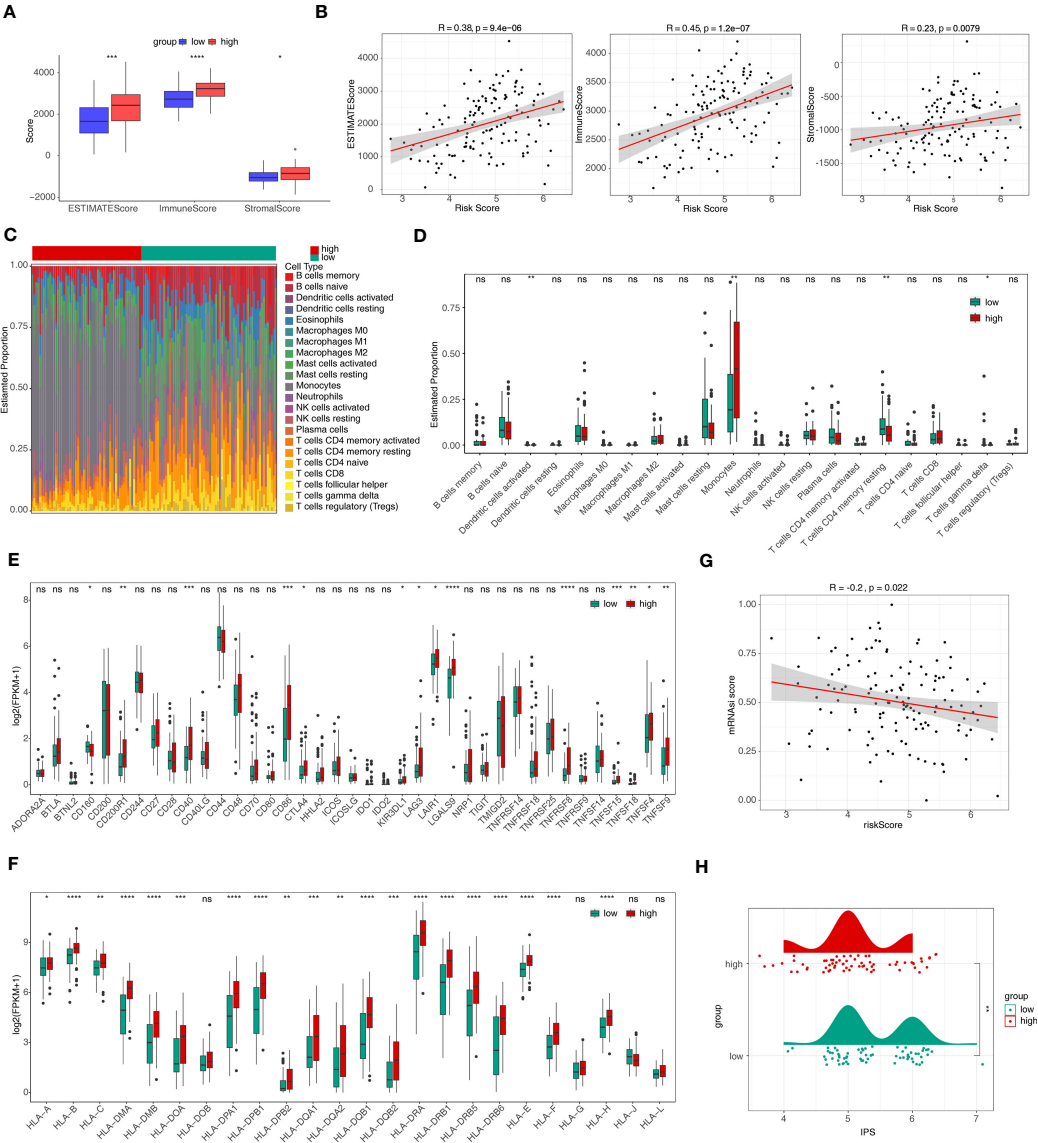
Immune infiltration and immune correlation analyses. **(A)** The discrepancies of immune score, stromal score, and estimate score between high- and low-risk groups. *p<0.05; ** p<0.01; ***p<0.001; ****p<0.0001. **(B)** The relevance of risk score to immune score, stromal score, and estimate score. **(C)** The heat map of abundance of 22 immune cells in two risk groups. **(D)** The discrepancies of immune cells between two risk groups. ns, not significant; *p<0.05; ** p<0.01. **(E, F)** The discrepancies of immune checkpoints **(E)** and HLA family genes **(F)** between high- and low-risk groups. ns, not significant; *p<0.05; ** p<0.01; ***p<0.001; ****p<0.0001. **(G)** The relevance of risk score and mRNAsi score. **(H)** Discrepancies of IPS between high- and low-risk groups. HLA, human leukocyte antigen; mRNAsi, stemness index based on mRNA expression; IPS, immunophenoscores. ns, not significant; *p<0.05; ** p<0.01; ***p<0.001; ****p<0.0001.

The ImmuneScore, StromalScore, and Estimate score of samples in the high-risk group were significantly higher than in the low-risk group (p < 0.05) (**Figure 6A**). **Figure 6B** revealed that ImmuneScore, StromalScore, and EstimateScore were positively associated with risk score. The proportion of 22 immune cell types was present in some abundance (**Figure 6C**) and 4 immune cell types (activated dendritic cells, monocytes, resting CD4 memory T cells, and gamma delta T cells) were significantly different between the two groups (**Figure 6D**). In addition, 14 immune checkpoints, 20 HLA family genes were significantly differentially expressed between the high/low-risk groups, and most factors were upregulated in the high-risk group (**Figures 6E, F**), and there was a negative correlation between risk score and mRNAsi score (R=-0.2 and p<0.05) (**Figure 6G**). Moreover, the IPS score was notably different between high- and low-risk groups, and the low-risk group was accompanied by higher score (**Figure 6H**). This showed that the prognostic risk model was linked to the immune microenvironment of AML, which provides some theoretical basis for immunotherapy of AML.

### 3.7 The differences of drug sensitivity between two risk groups

**Figure 7:**
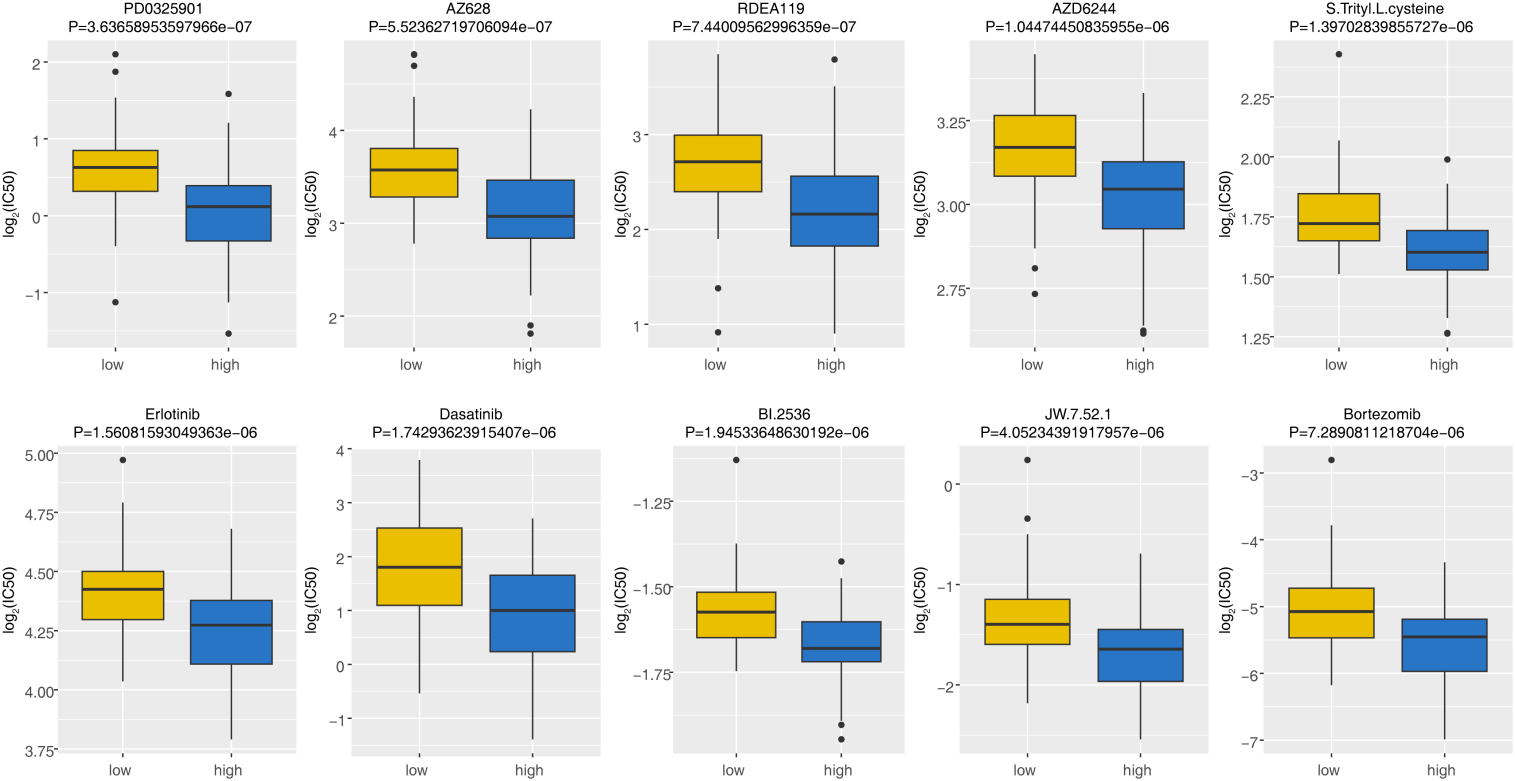
The discrepancies of drug sensitivity between high- and low-risk groups. The lower the log_2_(IC50) value, the more sensitive the groups of patient was to the drug. IC50, half-maximal inhibitory concentration.

IC50 was calculated for each AML patient in the two groups, yielding a total of 56 drugs with significantly different IC50s (**Supplementary Table 4**). **Figure 7** displayed box plots of the IC50 values for the top 10 significantly different treatment-sensitive drugs. The findings demonstrated that the low-risk group’s IC50 was much higher than the high-risk groups. Among them, Erlotinib, Dasatinib, BI.2536, and Bortezomib have been reported to be associated with the treatment of AML. Therefore, we believed that risk scores could be used to predict sensitivity to the above drugs for AML patients, where drug inhibitors might be more effective against organisms or cell lines in samples from high-risk group.

### 3.8 The expression of m6A-related ERGs by qRT-PCR

**Figure 8:**
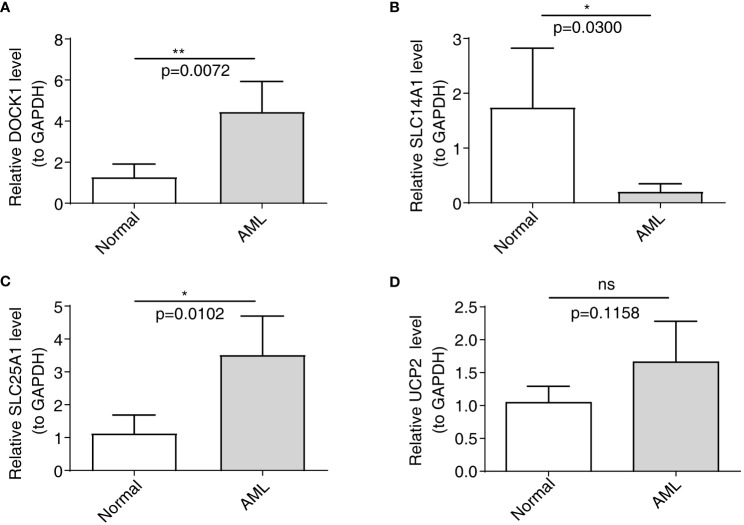
Validation of the expression of characterized genes by quantitative reverse transcriptase PCR. **(A)**: DOCK1; **(B)**: SLC14A1; **(C)**: SLC25A1; **(D)**: UCP2. ns, not significant; *p<0.05; **p<0.01.

The qRT-PCR analysis was performed to further verify characterized genes in AML and normal samples (**Table 2**; **Figures 8A–D**). The expression level of DOCK1 and SLC25A1 was higher in AML samples than in normal samples (**Figures 8A, C**). UCP2 mRNA expression was increased in AML patient samples as compare to healthy control samples, however the difference was not statistically significant (**Figure 8D**). The expression of SLC14A1 was higher in normal samples than in AML samples (**Figure 8B**).

**Table 2:** The results of qRT-PCR.

	Normal	AML	P value
DOCK1	1.2828 ± 0.6249	4.4652 ± 1.4656	0.0072
SLC14A1	1.7453 ± 1.0774	0.208. ± 0.1413	0.03
SLC25A1	1.1357 ± 0.5504	3.5250 ± 1.1734	0.0102
UCP2	1.0428 ± 0.2490	1.6593 ± 0.6231	0.1158

## 4 Discussion

The m6A methyltransferase METTL3 can impact the way AML is initiated and maintained. STM2457 is a high-efficiency selective first-in-class catalytic inhibitor specific to METTL3, and using it for tumor treatment can weaken the AML growth, and enhance the cell differentiation and apoptosis (36). In the study by Joselyn Cruz Cruz et al. (37), MerTK inhibition by small molecule tyrosine kinase, MRX2843, could change the leukemia microenvironment from tumor-permissive toward immune responsiveness to leukemia, as well as enhance the AML clearance mediated by immune. And the MerTK (or other vesicular cell receptors) on macrophages play an important role in mediating efferocytosis. m6A and genes related to efferocytosis can mediate the immune system, thereby affecting the AML development. Nevertheless, seldom studies have reported their joint roles. On that account, our study investigates m6A-related ERGs, aiming at contributing to new prognostic models and treatment strategies for AML patients.

In this study, we identified 7 m6A-DE-ERGs (CXCR4, PPARG, SGK1, WNK1, DNM1L, ADAM17, HIF1A), 27 miRNAs, and 7 lncRNAs, which were used to construct the lncRNA-miRNA-mRNA network. Some studies have reported the close association between SGK1, CXCR 4, PPARG, and ADAM17 and HIF1A and AML (38–42). DLEU1 can only exert impact on HIF1A through regulating hsa-miR-381-3p, while HIF1A can be impacted by various miRNAs. According to Abdul-Aziz AM et al. (42), PARP14 regulated the expression of HIF1A, thereby enhancing AML cell growth and glycolysis. On that account, applying miRNAs and 7 lncRNAs to regulate m6A-related DE-ERGs can impact the AML development.

Amino acids not only constitute proteins, but also serve as the intermediate metabolites for various biosynthetic pathways. The study by Yoko Tabe et al. (43) summed up the amino acid metabolism occurring in hematologic malignancy, and assisted in reclassifying amino acid-depleting enzymes into targeted therapeutic agents. In our study, amino acid metabolic pathways significantly impact AML development. According to Courtney L Jones et al. (44), the leukemia stem cell (LSC) population presented elevated amino acid uptake, steady-state levels, and catabolism, and drugs targeting LSC metabolic vulnerabilities could serve for eradicating LSCs in clinical practice. Hence, m6A-related DE-ERGs may regulate the AML occurrence and development through various amino acid metabolic pathways. Our study also reveals the close association between chemokine signaling pathway and AML. Chemokine refers to a family of small cytokines with chemotactic properties, consisted by 8-10 kilodaltons. Chemokine can traffic and regulate the proliferative, migratory, differentiative and homing activities of immune cells. The CXCR4 chemokine receptor can enhance the survival rate of various cell types. According to the study of Kimberly N Kremer et al. (45), CXCR4 chemokine receptor signaling regulated many of the Bcl-2 family members (Bcl-XL, Noxa, and Bak), thereby inducing AML cell apoptosis. Taken together, m6A-related DE-ERGs may be involved in AML progression by downregulating chemokine signaling pathway.

In the 14 m6A-related ERGs related-subtypes analysis, different subpopulations present obviously different monocytes, plasma cells and naive B cells, with more infiltrated in the worse prognosis cluster1. Clinical experiments in the study confirmed the significant role of monocytes, plasma cells and naive B cells in the AML pathogenesis, and its close association with myeloid tumor cell progression. Monocyte is the innate immune cell in the mononuclear phagocyte system and can remarkably regulate tumor development and progression. Plasma cell that secretes antibody serves as the core pillar of the humoral immunity, generated during the basic cellular restructuration from the naive B cells-antigen contact. When naive B cells are differentiated into extrafollicular B cells, antibody-secreting cells with weaker affinity and short life can be generated. Claire E Olingy et al. (46) made a comprehensive explanation of the monocyte heterogeneity in the homeostasis process, highlighted the role played by monocyte in the cancer development, as well as gave effective monocyte-targeted cancer treatment strategies. In the study by Maartje C A Wouters et al. (47), substantial evidences, by virtue of a comprehensive PubMed search, had proved that plasma cells positively impact the antitumor immunity, and it is suggested to enhance these responses in designing cancer immunotherapies. Helmink BA et al. (48) used mass cytometry for interrogating various surface proteins, finding the existence of naive B cells, memory B cells, activated memory B cells, and plasmablasts. Therefore, we speculated that different subtypes suggested the possible mediating roles played by monocytes, plasma cells and naive B cells in the prognosis of patients with different AML subtypes.

Our study constructed the prognosis model and the prognostic genes of UCP 2, DOCK 1, SLC14A1, and SLC25A1. Uncoupling protein 2 (UCP2), and mitochondrial uncoupling proteins belong to the family of mitochondrial anion carrier proteins (MACP). AML patients showed elevated UCP2 expression. Dongxu Gang et al. (49) found that UCP2 inhibition could lead to weakened AML cell line proliferation, cell cycle alternation, and apoptosis enhancement *in vitro*. Dedicator of cytokinesis 1(DOCK1) is the dedicator of cytokinesis proteins and the guanine nucleotide exchange factors specific to small Rho family G proteins. According to Sze-Hwei Lee et al. (50), highly expressed DOCK1 led to worse AML prognosis, and higher DOCK1 expression exhibited an obvious relevance to older age, higher platelet and peripheral blast counts, intermediate-risk cytogenetics, FLT3-ITD, MLL-PTD and PTPN11, NPM1, RUNX1, ASXL1 and DNMT3A mutations. Solute carrier family 14 member 1 (SLC14A1) is a gene that encodes a protein that mediates urea transport in erythrocytes (51). Through targeting SLC14A1, ARHGAP5 and PIK3CA, miR-10a-3p may be involved in the development of FLT3 mutation in adult AML (52). Solute carrier family 25 member 1(SLC25A1) is a mitochondrial carrier that facilitates the flow of citrate/isocitrate in mitochondria in exchange for the entry of malate in the cytoplasm (53, 54). According to a report, prognostic signature associated with SLC25A1 denotes AML patients worse prognosis (55). On that account, UCP 2, DOCK 1, SLC14A1, and SLC25A1 show important prognostic value in AML, but subsequent studies are still needed to explore their functions in AML.

The HLA correlation analysis revealed the highly expressed HLA in the high-risk group, which had a worse prognosis, and the two risk groups presented obvious difference in the expressions of the 14 immune checkpoints, 20 HLA family genes, and IPS. Immune checkpoint molecules, inhibitory and stimulatory, refer to ligand-receptor pairs that inhibit or stimulate the immune responses (56). Luca Vago et al. (57) conducted studies to test the latest immunotherapies for the specific targeting of AML cells (antibody therapy and cellular therapy, etc.) or the broader reactivation of antileukemia immunity (vaccines and checkpoint blockade, etc.), which combines complementary immunotherapeutic strategies with chemotherapeutics or other pharmacotherapies. Rikako Tabata et al. (58) demonstrated the underlying clinical benefits exhibited by immuno-oncology (IO) therapy specific to AML and ICIs with or without conventional chemotherapy. These prove the certain efficacy of immunotherapy in AML. Immune checkpoint, HLA and IPS are different in the two risk groups, suggesting their mediating roles in AML prognosis. Hence, the immune microenvironment of AML offers theoretical basis for immunotherapy of AML.

In the study, the predicted drugs are Erlotinib, Dasatinib, BI.2536, and Bortezomib, etc. Erlotinib, as a type of tyrosine kinase inhibitor, did not achieve a good response in AML patients in pilot study (59). Dasatinib is a type of kinase inhibitor, and has the function of inhibiting BCR-ABL, Src family kinases, c-Kit, and platelet-derived growth factor receptor kinase. Due to the inhibitory effect on BCR-ABL, it is usually used for treating chronic myeloid leukemia (CML) and Philadelphia chromosome-positive acute lymphoblastic leukemia. A subpopulation of AML patients show BCR-ABL expression. Patients with unselected AML present remarkably mixed clinical responses to dasatinib, which shall be validated in larger-scale studies (60). BI.2536 is a newly discovered Plk inhibitor capable of inducing mitotic arrest and apoptosis. According to the randomized, open-label, phase I/II trial, clinical activity in patients treated with single-agent BI 2536 provides the first evidence of the potential therapeutic value of targeted Plk in patients with relapsed refractory AML (61). Bortezomib, proteasome inhibitor, is the mainstream drug for treating various myeloma and mantle cell lymphoma. AML patients give a series of different clinical responses to the chemotherapy regimens that combine bortezomib, and some cases show a complete remission rate over 80% (62). On these account, dasatinib, BI.2536, and bortezomib may be applicable for treating AML, which shall be more deeply concerned in future studies.

In summary, using bioinformatic methods, this study has identified prognostic genes for AML and constructed a prognostic model associated with m6A and ERGs. Differential analyses were conducted between high and low-risk groups, evaluating immune cell infiltration, immune therapy response, functional enrichment, and drug sensitivity. However, the study has discernible limitations. The analysis predominantly relies on a constrained number of samples from public databases, highlighting the imperative need for an expanded sample size. While gene expression levels have been validated through qRT-PCR, the requisite further verification and elucidation of potential molecular mechanisms necessitate animal experiments. Moreover, the analyses pertaining to immune therapy and drug sensitivity in the study require clinical validation to ascertain their clinical value further. Such exploration will constitute the main thrust of our ensuing research efforts. Ultimately, our findings furnish researchers with a novel theoretical framework for delving deeper into the relationship between m6A regulatory factors, efferocytosis, and AML, thereby providing new targets for enhancing the prognosis and treatment of AML.

## Data availability statement

The datasets presented in this study can be found in online repositories. The names of the repository/repositories and accession number(s) can be found in the article/**Supplementary Material**.

## Ethics statement

The studies involving humans were approved by Seventh Affiliated Hospital, Sun Yat-Sen University. The studies were conducted in accordance with the local legislation and institutional requirements. The participants provided their written informed consent to participate in this study. The manuscript presents research on animals that do not require ethical approval for their study.

## Author contributions

YW: Validation, Writing – original draft. TB: Resources, Writing – review & editing. JT: Conceptualization, Data curation, Writing – review & editing. X-JX: Formal analysis, Writing – review & editing. CL: Project administration, Software, Writing – original draft. BL: Funding acquisition, Investigation, Writing – review & editing. T-TS: Methodology, Visualization, Writing – review & editing.

## Supplementary material

Supplementary Figure 1 The expression of prognostic genes. **(A, B)** The discrepancies of the expression of four prognostic genes in the GSE9476 **(A)** and GSE13159 **(B)** datasets. *p<0.05; ** p<0.01; ***p<0.001; ****p<0.0001.

Supplementary Table 1 The clinical information of AML and normal samples in qRT-PCR.

Supplementary Table 2 27 miRNAs targeting 14 m6A-associated DE-ERGs.

Supplementary Table 3 7 lncRNAs targeting miRNAs.

Supplementary Table 4 The IC50 value for 138 drugs in patients in the high and low-risk groups.
