# Supplementary figures and images for: Construction of an acute myeloid leukemia prognostic model based on m6A-related efferocytosis-related genes
Source: Front Immunol. 2023 Nov 22;14:1268090. doi: 10.3389/fimmu.2023.1268090 (PMC10704160; doi:10.3389/fimmu.2023.1268090)

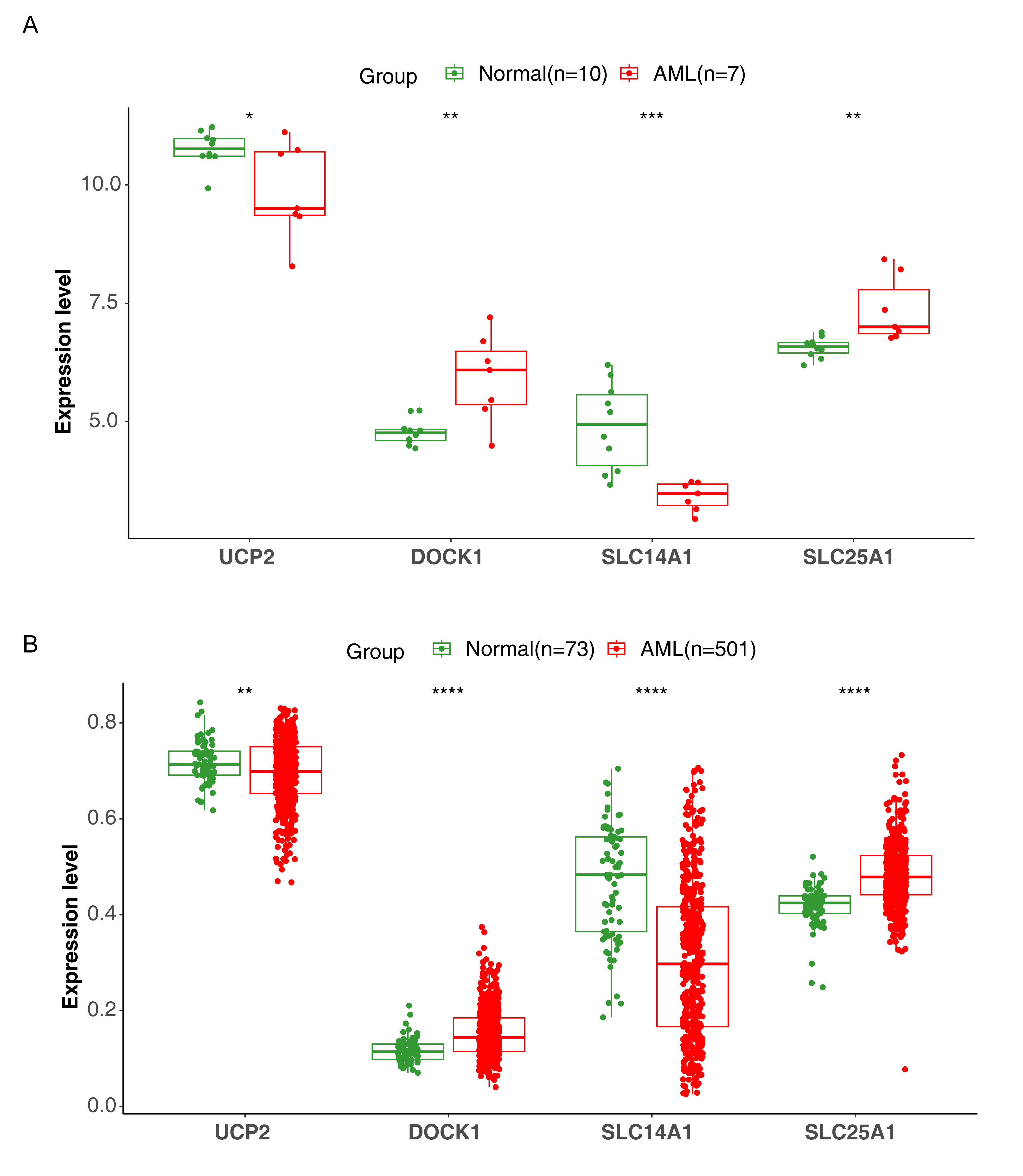

Supplement: Supplementary Figure 1 — The expression of prognostic genes. (A, B) The discrepancies of the expression of four prognostic genes in the GSE9476 (A) and GSE13159 (B) datasets. *p<0.05; ** p<0.01; ***p<0.001; ****p<0.0001. [file Image_1.tif]
